# The role of transformational leadership, job demands and job resources for patient safety culture in Norwegian nursing homes: a cross-sectional study

**DOI:** 10.1186/s12913-020-05671-y

**Published:** 2020-08-26

**Authors:** Camilla Seljemo, Petter Viksveen, Eline Ree

**Affiliations:** SHARE – Centre for Resilience in Healthcare, Faculty of Health Sciences, University of Stavanger, Stavanger, Norway

**Keywords:** Transformational leadership, Nursing homes, Patient safety culture, Patient safety, Job demands, Job resources

## Abstract

**Background:**

Transformational leadership style is considered to be of importance to increase patient safety, to facilitate a balance between job resources and job demands, and to create a sound patient safety culture within health care services. However, there is limited research assessing these associations within the context of nursing homes. The aim of this study was to assess the association between transformational leadership, job demands and job resources; and patient safety culture and employees’ overall perception of patient safety in nursing homes.

**Method:**

A cross-sectional survey of employees in four Norwegian nursing homes was conducted (*N* = 165). Multiple hierarchical regression analysis was used to assess the explained variance of transformational leadership, job demands and job resources on patient safety culture and overall perception of patient safety.

**Results:**

Transformational leadership explained 47.2% of the variance in patient safety culture and 25.4% of overall perception of patient safety, controlling for age and gender (*p* < 0.001). Additionally, job demands and job resources explained 7.8% of patient safety culture and 4.7% of overall perception of patient safety (*p* < 0.001).

**Conclusion:**

Implementing transformational leadership style may be important in creating and sustaining sound patient safety culture in nursing homes. Furthermore, leaders should make an effort to facilitate a good work environment with an optimal balance between job demands and job resources, as this in turn might have a positive influence on patient safety culture.

## Background

Patient safety and patient safety culture has in recent years been the subject of extensive research, but most of the research has been carried out in hospitals [1]. Mortality rates due to adverse medical effects are 20 times higher for older patients (70 years+), compared to those who are younger (15–49 years) [2]. Improving patient safety measures within the health care services is therefore particularly important for the elderly. A retrospective record review estimated that over 70% of adverse events in home care are preventable [3].

An increasing number of older people are in need of long-term health care services outside the hospital setting due to medical advances and decreasing disease-related mortality [4, 5]. People live longer, though often with chronic diseases or symptoms causing health loss [6]. An increasing proportion of older people is and will continue to be in need of care and treatment in the coming years, resulting in increased pressure on nursing homes. This means that we face new challenges related to the complexity of the health care system, which in turn increases the risk of adverse events in health care organisations [7].

Focus on patient safety increased following the publication of two reports; “To err is human- building a safer health system” by the American Institute of Medicine [8], and “An organisation with a memory” by the UK Department of Health [9]. The reports highlighted the importance of organisational culture as part of the key strategies required to strengthen patient safety. There is no simple answer to the question of how health care can become safer, but safety culture is considered a key area of focus for the coming years [7, 10]. Moreover, the results of several studies suggest that there may be a positive relationship between patient safety culture and perceived working conditions, and positive objective patient outcomes such as reduction in the frequency of falls and reduced mortality rates, as well as increased patient satisfaction [11, 12].

Patient safety culture can be defined as “*the product of individual and group values, attitudes, perceptions, competencies, and patterns of behaviour that determine the commitment to, and the style and proficiency of, an organisation’s health and safety management. Organisations with a positive safety culture are characterised by communications founded on mutual trust, by shared perceptions of the importance of safety and by confidence in the efficacy of preventive measures”* [13]. The US National Patient Safety Foundation (NPSF) emphasizes leaders’ role in establishing and sustaining a patient safety culture as an important focus area in patient safety work [14]. Patient safety culture and leadership was highlighted as fundamental for improving patient safety when the Norwegian patient safety program “In safe hands 24-7” was established in 2011 [15]. This was followed by new regulations implemented in January 2017 by the Norwegian Government, emphasizing leaders’ responsibility to establish a sound patient safety culture as a part of the overall aim to prevent harm [16]. Leaders’ role in creating a culture of safety and in patient safety work overall has long been considered to be of key importance to patient safety [17, 18]. According to McFadden [19], creating a culture of safety begins with leadership, and transformational leadership is directly associated with creating and sustaining a safety culture. Recent research also shows that transformational leadership is a significant predictor for patient safety culture in Norwegian home care services [20]. The four components characterizing transformational leaders are: idealized influence, inspirational motivation, intellectual stimulation and individualized consideration [21]. These leadership characteristics contribute to enabling employees to act beyond their own needs and interests, for the greater good of the whole group. The transformational leader will also identify the organizational needs and work to transform the organizational culture, to the benefit of the organization itself [18]. Based on theory and previous research, we hypothesize that transformational leadership is positively associated with higher levels of patient safety culture and overall perception of patient safety. Transformational leadership has also been suggested to positively affect health personnel’s work environment [22]. According to the Job-Demands Resources (JD-R) model by Bakker and Demerouti [23, 24], high levels of job demands lead to increased risk of burnout. On the other hand, employees may keep up motivation and reduce risk of burnout if they have sufficient resources in addition to a high degree of job demands. Bakker and Demerouti specifically mention the supervisory relationship and supportive feedback as important resources. The results of previous studies suggest that work environmental factors related to job demands and job resources influence patient safety outcomes [12, 22, 25]. Based on the job-demands resources theory and previous literature, we hypothesize that job demands are negatively associated with patient safety culture and overall perception of patient safety. Furthermore, we hypothesize that higher scores on job resources are positively associated with patient safety culture and overall perception of patient safety.

Managers play an important role in balancing job demands and job resources within the workplace [26]. Research suggests that a transformational leadership style positively influences nurses’ job satisfaction, work engagement and the psychosocial work environment, which also reduces the occurrence of adverse events in clinical practice [22, 27–29]. Adopting a transformational leadership style can also reduce factors related to burnout in nurses [30, 31]. It can prevent them from quitting their jobs and increase staff well-being and the quality of care. The American Institute of Medicine [32] recommends leaders to adopt a transformational leadership style in the process of creating optimal working conditions for nurses, which is essential to prevent accidents and improve patient safety. We therefore examine the extent to which job demands and job resources may explain the variance in patient safety culture and overall perceptions of patient safety beyond the impact of transformational leadership.

There is limited research on patient safety culture in nursing homes, especially in Europe [1]. One previous study assessed the association between transformational leadership, job demands and job resources, and their impact on patient safety culture in Norwegian home care services [20]. However, no previous studies have explored these associations in nursing homes. The aim of this study was to assess the association between transformational leadership, job resource and job demand scores; and patient safety culture and employees’ overall perception of patient safety in nursing homes.

## Methods

A cross-sectional design was used.

### Sample

The sample consisted of 165 nursing home employees with different professional backgrounds. The majority were female (92%) aged 50 to 59 (30%), and 46% had been working in nursing homes for more than 10 years (Table 1).

**Table 1:** Respondents background demographic information

Age	N (%)	Position/education	N (%)	Years of employment	N (%)
20–29	20 (12.1)	Managerial positions	11 (6.7)	Less than 1 year	20 (12.1)
30–39	39 (23.6)	Min. 3-year education	67 (40.6)	1–5 years	41 (24.8)
40–49	26 (15.8)	High school education	79 (47.9)	6–10 years	27 (16.4)
50–59	50 (30.3)	Assistant (unskilled)	3 (1.8)	11–15 years	24 (14.5)
60 +	30 (18.2)	Administrative personnel	2 (1.2)	16–20 years	20 (12.1)
		Other	3 (1.8)	21 years +	33 (20)

### Data collection

Four different nursing homes in southwestern Norway participated in the study. A total of 254 healthcare practitioners including unit and department managers at first line level with personnel responsibilities were invited by email to participate in the survey in the spring of 2018. An online questionnaire resulted in a response rate of 65% (*N* = 165). Co-researchers from the Development Centres of Nursing Home and Home Care Services facilitated recruitment by contacting nursing home managers in each participating nursing home. The selection strategy was purposive, to include a variety of nursing homes with different locations (urban and rural), and with variation in municipality and unit size. Inclusion criteria were: being employed in minimum 30% positions and being able to read Norwegian.

### Survey instruments

The questionnaire consisted of several validated scales used to measure patient safety culture, transformational leadership, job demands and job resources, as well as background characteristics such as age, gender, occupational status, and years of employment. The scales were selected due to their suitability to the target group, feasibility (e.g., time to respond), and availability of Norwegian validated versions.

#### Outcome variables

*The patient safety culture* was measured using the validated Norwegian version of the Nursing Home Survey on Patient Safety Culture (NHSOPSC) [33]. This version contains a 10-factor model, which has been found to be suitable for Norwegian nursing home settings [33]. The scale was chosen for this study since it is designed specifically to assess patient safety culture within the context of nursing homes, and its Norwegian version has been validated.

The scale contains 42 items rated on 5-point Likert scales (from 1“totally disagree” to 5 “totally agree”). In the current study, the mean score of all 42 items was used as an outcome measure of patient safety culture. In addition, we used one of the instrument’s general questions to assess healthcare practitioners’ overall perception of patient safety in the nursing home, “Please give this nursing home an overall rating on patient safety”. Cronbach’s Alpha for the 42 items was .93.

#### Predictor variables

*Transformational leadership* was measured using The Global Transformational Leadership Scale (GTL) [34] where participants are asked to rank their leader’s behavior on 5-point Likert scales (from 1 “never” to 5 “very often”) on seven items. The scale was chosen for this study since it is well established and previous research suggested good internal consistency [34, 35]. Moreover, it measures the components that are considered essential for a transformational leader and it has previously been used to assess transformational leadership within a broad range of Norwegian work settings [35]. Examples of items include “Gives encouragement and recognition to staff” and “Fosters trust, involvement and cooperation among team members”. In the current study, Cronbach’s Alpha was .95.

*Job demands and job resources* was measured using the Short Inventory to Monitor Psychosocial Hazards (SIMPH) scale [36], which is in line with the Job Demands-Resources model by Bakker and Demerouti [23]. The instrument contains 24 items that measure job demands and job resources on 4-point Likert scales (from 1 “never” to 4 “always”). Job demands consist of the following three dimensions: 1) “pace of work” (four items); 2) “mental workload” (three items); and 3) “emotional workload” (five items). Examples of items include: “Do you work under time constraints?” (pace of work), “Are there many things to remember in your job?” (mental workload), and “Is your work heavy from an emotional point of view?” (emotional workload). For the three dimensions in job-demands, Cronbach’s Alpha was .91 (pace of work), .74 (mental workload) and .75 (emotional workload) respectively. Job resources consist of the following three dimensions: 1) “skill utilization” (four items); 2) “autonomy” (four items); and 3) “participation” (five items). Item examples include: “Do you learn new things in your work?” (skill utilization), “Do you have an influence on the pace of work?” (autonomy), and “Can you participate in decisions that affect areas of your work?” (participation). Cronbach’s Alpha for the three dimensions in job-resources was .76 (skill utilization), .72 (autonomy) and .75 (participation).

### Analysis

The associations between the variables in the study were explored using Pearson’s correlation analysis. Hierarchical multiple regression analyses were used to examine the extent to which transformational leadership, job demands, and job resources predict the outcome variables patient safety culture and overall perception of patient safety. This analysis was considered the most appropriate statistics to test our hypotheses, as it allows us to explore the predictive value of all dimensions in total (the whole model), as well as the relative contribution of each dimension [37]. Alpha was set to .05. No violations of normality, linearity, multicollinearity and homoscedasticity were found. We had no missing values in the data set, since respondents had to answer each question before proceeding to the next. Statistical analyses were carried out using SPSS (version 25.0).

## Results

### Correlation between variables

Transformational leadership, patient safety culture, overall perception of patient safety and job resources (skill utilization, autonomy, participation) were all positively correlated (*p* < 0.001). Work pace and emotional workload were both negatively correlated with patient safety culture, overall perception of patient safety and transformational leadership (Table 2). However, the mental workload dimension did not correlate significantly with either patient safety culture, overall perception of patient safety or transformational leadership and was therefore omitted from the regression analyses to avoid spurious regression.

**Table 2:** Correlation between the variables using Pearson’s correlation coefficient (r)

Variables	M	SD	1	2	3	4	5	6	7	8	9
1 PSC^a^	3.75	.51	1								
2 TFL^b^	3.57	.81	.679**	1							
3 Work Pace	2.38	.64	−.329**	−.224**	1						
4 Mental workload	2.99	.58	.007	−.015	.528**	1					
5 Emotional workload	1.99	.43	−.332**	−.212**	.333**	.272**	1				
6 Skill utilization	2.80	.57	.478**	.465**	−.071	.213**	−.233**	1			
7 Autonomy	2.53	.54	.347**	.327**	−.200*	.036	−.203**	.360**	1		
8 Participation	2.54	.66	.484**	.529**	−.213**	.016	−.321**	.579**	.554**	1	
9 Overall PS^c^	4.06	.83	.667**	.501**	−.231**	−.015	−.273**	.257**	.244**	.278**	1

** Correlation is significant at the .01 level (2-tailed) * Correlation is significant at the .05 level (2-tailed) ^a^*PSC* Patient safety culture, ^b^*TFL* Transformational leadership, ^c^*PS* Patient safety

### Hierarchical regression of patient safety culture

In the hierarchical regression analysis of patient safety culture (Table 3), the transformational leadership model explained 47.2% of the variance when controlling for age and gender (*F* change = 138.6, *p* < 0.001*).* Job demands (work pace, emotional workload) and job resources (skill utilization, autonomy, participation) explained an additional 7.8% of the variance in patient safety culture (*F* change = 5.4, *p* < 0.001.). The final model explained 55.0% of the variance and transformational leadership was the strongest predictor for patient safety culture (*β* = .522, *p* < 0.001), followed by skill utilization (*β* = .166, *p* < 0.05), work pace (*β* = −.135, *p* < 0.05) and emotional workload (*β* = −.126, *p* < 0.05).

**Table 3:** Hierarchical regression analysis for variables predicting patient safety culture (*N* = 165)

Variable	Model 1			Model 2			Model 3		
	β	B	SE B	β	B	SE B	β	B	SE B
Age	.113	.045	.031	.101	.040	.023	.060	.024	.022
Gender	.083	.158	.050	−.012	−.023	.111	.010	.018	.110
Transformational leadership				.681**	.434	.037	.522**	.333	.042
Work Pace							−.135*	−.108	.048
Emotional workload							−.126*	−.149	.072
Skill Utilization							.166*	.149	.061
Autonomy							.052	.050	.063
Participation							.010	.008	.062
*R*^*2*^		.018			.472			.550	
*R*^*2*^ Change		.018			.455**			.078**	

*p < .05 **p < .001

### Hierarchical regression of overall perception of patient safety

In the hierarchical regression analysis of overall perception of patient safety (Table 4), the transformational leadership model explained 25.4% of the variance when controlling for age and gender (*F* change = 51.8, *p* < 0.001*).* Adding job demands and job resources increased the explained variance with 4.7% (*F* change = 2.1, *p* > 0.05), but did not result in a significant change to the final model. Total explained variance of the final model was 30.1%. In the final model, transformational leadership was the strongest predictor (*β* = .447, *p* < 0.001), and the only additionally significant predictor was emotional workload (*β* = −.165, *p* < 0.05*)*.

**Table 4:** Hierarchical regression analysis for variables predicting overall perception of patient safety (*N* = 165)

Variable	Model 1			Model 2			Model 3		
	β	B	SE B	β	B	SE B	β	B	SE B
Age	−.026	−.016	.050	−.055	−.022	.043	−.055	−.035	.044
Gender	.111	.343	.242	.043	.131	.213	.072	.221	.220
Transformational leadership				.495**	.508	.070	.447**	.459	.085
Work Pace							−.096	−.124	.096
Emotional workload							−.165*	−.314	.144
Skill Utilization							.036	.052	.123
Autonomy							.078	.119	.127
Participation							−.104	−.131	.124
*R*^*2*^		.014			.254			.301	
*R*^*2*^ Change		.014			.240**			.047	

*p < .05 **p < .001

## Discussion

The results of this study showed that transformational leadership was a strong predictor of patient safety culture and overall perception of patient safety. Job demands and job resources were also important for the outcomes, although with some differences regarding which specific factors were most significant.

The findings are in line with a recent study in the Norwegian home care setting [20], showing that transformational leadership was a strong predictor for patient safety culture. Similar to the current study, the job demand ‘work pace’ negatively predicted patient safety culture and job resources were significant predictors in both studies [20].

Due to the complexity of the rapidly changing health care services, there is a need for increased knowledge and skills to ensure the safety of patients. Skill utilization in Norwegian nursing homes seems to be an important factor for patient safety culture. To build and develop skills, there is a need for leaders who are open to receive feedback from frontline workers, in order to expand their understanding and gain an overview of the challenges they are facing in daily care. Transformational leaders are capable of seeing employees’ individual needs and keep them intellectually stimulated, which might impact employees’ willingness to acquire new knowledge and develop new skills. Previous research shows that there is an indirect link between safety performance and transformational leadership through knowledge-related job characteristics [38]. Skills and knowledge are also prioritised areas of focus in patient safety work in the coming years [7, 39].

The fact that transformational leadership is a stronger predictor than job demands and resources, might be a result of transformational leadership also having a positive effect on the psychosocial work environment. Several other studies found that transformational leadership has a positive effect on healthcare personnel’s job satisfaction [27, 29, 40], which seemed to decrease the occurrence of adverse events [22] and improve the quality of care [31]. This is also supported by another study which found that transformational leadership positively influenced psychosocial resources and thereby made job demands more manageable [41]. Supervisor support and a high-quality relationship with the supervisor are suggested to be particularly important resources, enabling employees to cope with high job demands [24, 42, 43]. The transformational leader has the ability to provide individual support and see the employee’s individual needs.

The results of this study indicate that transformational leadership seems to contribute to improve patient safety culture and patient safety in nursing homes. This also supports national guidelines focusing on management as essential for patient safety culture [15, 39], and the key role of managers in building a safety culture has also been highlighted in recent reports and regulations [7, 14, 16, 39].

The importance of transformational leadership in creating a patient safety culture might be a key element at a time where healthcare services are continuously changing, and it takes time to implement new regulations and professional practices. Understanding how to build safety barriers due to these change processes is necessary for nursing managers in order to sustain a safety culture [44]. Being a transformational leader implies being able to adapt to every situation and contribute to change the current work culture to improve patient safety. However, Tafvelin, Isaksson and Westerberg [45] found that middle managers who wanted to practice a transformational leadership style, experienced that the top management was an obstacle to change due to lack of support and limited influence. This may indicate that not only the middle managers need to adapt their leadership style, but managers and leaders at every level in an organization should do so. Creating a safety culture requires knowledge of how systems and people interact, and a broader perspective to understand and act upon the multiple factors that influence the safety culture and psychosocial work environment [46].

### Strengths and limitations

This study has several methodological strengths and limitations. It is the first study investigating the association between transformational leadership, patient safety culture and overall perception of patient safety in nursing homes. Therefore, this study provides new knowledge about factors that might contribute to improve patient safety culture and patient safety in the nursing home setting.

An average score for the patient safety culture was used, which means that we do not know exactly which factors in the culture are most decisive. However, the mean score was used due to high correlation and overlap between some of the dimensions in NHSOPSC, and because the purpose of the study was to examine the role of transformational leadership, job demands and job resources on the total patient safety culture. We recommend that future studies explore the contribution of transformational leadership, job demands and job resources on the different dimensions of patient safety culture.

Overall perception of patient safety was measured using a single item. However, the item correlated strongly with related concepts, and has been recognized as reliable outcome measure in previous studies [33, 47]. It is important to be aware that the responses on this outcome only reflect employees’ perception of patient safety and not the actual status of patient safety in the nursing homes. Primary health care practitioners are not required to routinely collect data on adverse events in Norway. However, we recommend that objective outcomes of adverse events are included in future studies.

The cross-sectional design, the relatively small sample and the low response rate limits the generalizability of the findings. However, the heterogeneity of the included units (small and large units and municipalities, urban and rural) are representative for the variability present between nursing homes across the country. Furthermore, the response rate was similar to other Norwegian nursing home studies [48, 49], indicating a general challenge in obtaining high response rates in this setting. The small sample size did not allow for sub-group analyses with different professions, age groups, years of experience or between departments within each nursing home. Thus, future studies should explore whether the associations differ between different groups of employees, using a longitudinal design. Another option for future research could be to examine whether job demands and resources mediate the effect of transformational leadership on patient safety culture and overall perceptions of patient safety. Due to the small sample size we could not justify conducting valid path analyses of mediation and moderation effects in this study [50]. Structural equation modelling could be used to further assess the mechanisms through which transformational leadership affects patient safety culture.

## Conclusion

This study shows that transformational leadership is a significant and positive contributor to patient safety culture and overall perceptions of patient safety in nursing homes. Job demands and the job resource ‘skill utilization’ are also associated with the patient safety culture. The findings indicate that leaders have a key role in creating and maintaining a sound patient safety culture and improving patient safety. Leaders’ efforts in facilitating a good work environment with an optimal balance between job demands and job resources might in turn have a positive impact on the patient safety culture. Leaders should acquire knowledge about the transformational leadership style and implement it as a strategy in their overall quality and patient safety improvements efforts.

## Data Availability

The dataset is available on request from the corresponding author.

## Abbreviations

NHSOPSC: Nursing Home Survey on Patient Safety Culture
GTL: Global Transformational Leadership Scale
SIMPH: Short Inventory to Monitor Psychosocial Hazards
PSC: Patient Safety Culture
PS: Patient Safety
TFL: Transformational leadership
